# Transplantation of a kidney with a heterozygous mutation in the *SLC22A12* (URAT1) gene causing renal hypouricemia: a case report

**DOI:** 10.1186/s12882-020-01940-4

**Published:** 2020-07-16

**Authors:** Kiyokazu Tsuji, Mineaki Kitamura, Kumiko Muta, Yasushi Mochizuki, Takayasu Mori, Eisei Sohara, Shinichi Uchida, Hideki Sakai, Hiroshi Mukae, Tomoya Nishino

**Affiliations:** 1 Department of Nephrology, Nagasaki University Graduate School of Biomedical Sciences, Nagasaki, Japan; 2 Department of Urology, Nagasaki University Graduate School of Biomedical Sciences, Nagasaki, Japan; 3 Department of Nephrology, Graduate School of Medical and Dental Sciences, Tokyo Medical and Dental University, Tokyo, Japan; 4 Department of Respiratory Medicine, Nagasaki University Graduate School of Biomedical Sciences, Nagasaki, Japan

**Keywords:** SLC22A12, Renal hypouricemia, Chimerism, Renal allografts, Fluorescence in situ hybridization

## Abstract

**Background:**

Renal hypouricemia (RHUC) is a genetic disorder caused by mutations in the *SLC22A12* gene, which encodes the major uric acid (UA) transporter, URAT1. The clinical course of related, living donor-derived RHUC in patients undergoing kidney transplantation is poorly understood. Here, we report a case of kidney transplantation from a living relative who had an *SLC22A12* mutation. After the transplantation, the recipient’s fractional excretion of UA (FEUA) decreased, and chimeric tubular epithelium was observed.

**Case presentation:**

A 40-year-old man underwent kidney transplantation. His sister was the kidney donor. Three weeks after the transplantation, he had low serum-UA, 148.7 μmol/L, and elevated FEUA, 20.8% (normal: < 10%). The patient’s sister had low serum-UA (101.1 μmol/L) and high FEUA (15.8%) before transplant. Suspecting RHUC, we performed next-generation sequencing on a gene panel containing RHUC-associated genes. A heterozygous missense mutation in the *SLC22A12* gene was detected in the donor, but not in the recipient. The recipient’s serum-UA level increased from 148.7 μmol/L to 231.9 μmol/L 3 months after transplantation and was 226.0 μmol/L 1 year after transplantation. His FEUA decreased from 20.8 to 11.7% 3 months after transplantation and was 12.4% 1 year after transplantation. Fluorescence in situ hybridization of allograft biopsies performed 3 months and 1 year after transplantation showed the presence of Y chromosomes in the tubular epithelial cells, suggesting the recipient’s elevated serum-UA levels were owing to a chimeric tubular epithelium.

**Conclusions:**

We reported on a kidney transplant recipient that developed RHUC owing to his donor possessing a heterozygous mutation in the *SLC22A12* (URAT1) gene. Despite this mutation, the clinical course was not problematic. Thus, the presence of donor-recipient chimerism in the tubular epithelium might positively affect the clinical course, at least in the short-term.

## Background

Renal hypouricemia (RHUC) is a genetic disorder caused by a defect in the urate transporter 1 (URAT1) protein, often causing urolithiasis and exercise-induced acute renal failure [1–5]. URAT1 is responsible for uric acid (UA) reabsorption at the brush border membrane of the renal proximal tubules and is encoded by the *SLC22A12* gene [4, 5]. In addition, a heterozygous missense mutation in *SLC22A12* can cause RHUC [6].

At present, two reports showed successful kidney transplantation from living, related donors and recipients affected with hereditary RHUC [7, 8]. However, whether the RHUC donor affects the outcome of the recipient remains unknown. Therefore, the clinical course of these cases should be carefully evaluated. The role of chimeric cells in graft adaptation has long been the subject of transplantation research. The occurrence of sex chromosome chimerism, particularly Y chromosome chimerism, is commonly observed in sex-mismatched kidney transplants through renal allografts performed 1 year or more post-transplant [9]. The presence of Y chromosomal tubular chimerism is generally detected with fluorescence in situ hybridization (FISH) or chromogenic in situ hybridization methods [9].

We reported on a case of donor-derived RHUC in a kidney recipient, focusing on the detection of Y chromosome chimerism in the tubular epithelium of the recipient using FISH.

## Case presentation

The recipient was a 40-year-old Japanese man that was diagnosed with proteinuria during a previous medical examination at 28 years of age. However, he did not receive any form of treatment for this condition and had not received any kind of medical care until 38 years of age, when he sought treatment for severe fatigue and anorexia. Laboratory tests showed a serum creatinine (S-Cr) level of 1113.8 μmol/L and serum-UA (S-UA) level of 743.5 μmol/L. He was referred to the nephrology department of another hospital to receive hemodialysis. The etiology of his renal failure was unknown because a renal biopsy was not performed.

Two years later, he underwent kidney transplantation. The patient’s donor was his living sister, and the clinical course after transplantation was uneventful. Three weeks after kidney transplantation, his S-Cr was 100.7 μmol/L, and he had a slightly low S-UA (148.7 μmol/L; normal: 220–463.9 μmol/L) with a fractional excretion of UA (FEUA) of 20.8% (normal: < 10%). A physical examination of the patient did not reveal any abnormalities.

The donor was the patient’s 42-year-old sister. Before donation, her S-UA and FEUA levels were 101.1 μmol/L and 15.8%, respectively. Other clinical data were normal (Table 1). There was no history of kidney stones, acute renal failure, and/or hypouricemia. Their father was alive and healthy, and his medical history did not indicate any kidney dysfunction, including urolithiasis and acute renal failure. However, their mother had two episodes of urolithiasis at 19 years of age.

**Table 1:** Laboratory data of the donor, before kidney transplantation

	Normal ranges for female	
**Complete blood cell count**		
WBC (× 10^9^ cells/L)	3.3–8.6	4.5
RBC (× 10^12^ cells/L)	3.86–4.92	4.47
Hemoglobin (g/L)	116–148	108
Hematocrit (%)	35.1–44.4	34.9
Platelets (×10^9^/L)	158–348	329
**Serum biochemistry**		
Total protein (g/L)	66–81	70
Albumin (g/L)	41–51	41
BUN (mmol/L)	2.85–7.14	4.28
Cr (μmol/L)	40.66–69.83	44.25
Uric acid (μmol/L)	154.64–327.14	101.1
Sodium (mmol/L)	138–145	139
Potassium (mmol/L)	3.6–4.8	4.2
Chloride (mmol/L)	101–108	105
Calcium (mmol/L)	2.19–2.51	2.19
Phosphorous (mmol/L)	0.87–1.48	0.9
**Urinalysis**		
pH	4.6–7.5	6.0
Specific gravity	1.006–1.022	1.007
Protein		(−)
Occult blood		(−)
RBC sediment (/HPF)		1–4
WBC sediment (/HPF)		1–4
CCr (mL/min)		260
Urinary protein excretion (mg/day)		50
Urine Cr (μmol/L)		3500.6
Urine uric acid (μmol/L)		1266.9

*BUN* blood urea nitrogen, *Cr* creatinine, *CCr* creatinine clearance, *RBC* red blood cells, *WBC* white blood cell, (−), not detected

Based on these findings, we suspected that the donor had RHUC. After obtaining the informed consent from both the donor and the recipient, we performed next-generation sequencing with a gene panel containing RHUC-associated genes on both of them, including *SLC22A12*, the most common causative gene of RHUC and *SLC2A9* [10]*.* We found a missense heterozygous G to A substitution in position 269 in the exon 1 (NM_144585.4:c.269G > A (p.Arg90His)) in the donor, but not in the recipient. The American College of Medical Genetics and Genomics variant classifications for this variant are shown in Supplemental Material 1 [11].

**Fig. 1:**
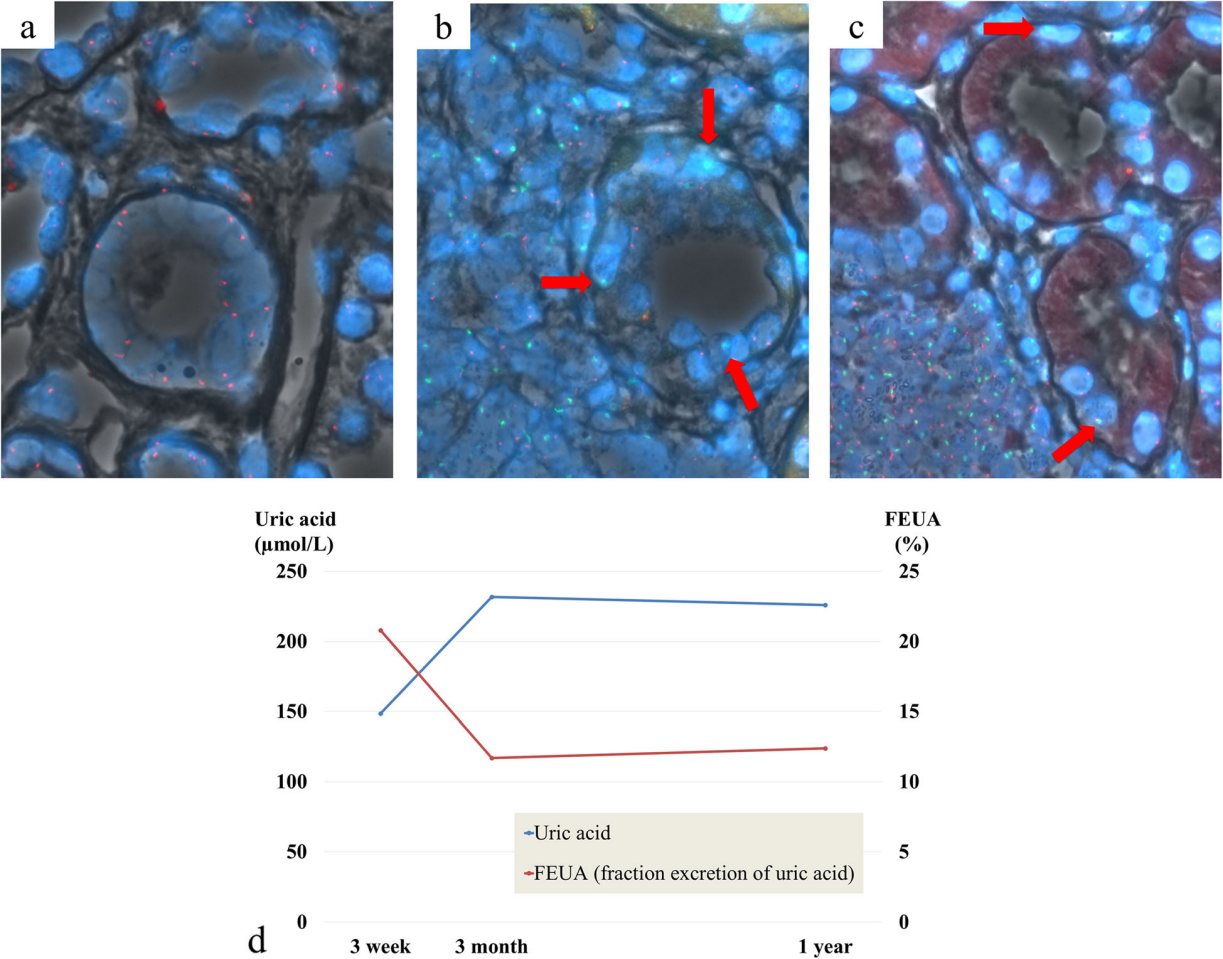
**a** 0-h biopsy; FISH with a probe against X chromosome (red) of the normal tubular epithelium. **b**, **c** 3-month protocol biopsy; FISH with probes against the Y chromosome (green) and the X chromosome (red) of the recipient tubular epithelium. Sex chromosomes are indicated with an arrow. **d** Clinical course; The levels of uric acid increased after transplantation; on the other hand, the fractional excretion of uric acid decreased after transplantation. FEUA: fractional excretion of uric acid

The recipient had an excellent transplantation outcome: two years after the surgery, he had no major complications, including urolithiasis or exercise-induced acute renal failure. The recipient’s S-UA level was 231.9 and 226.0 μmol/L, 3 months and 1 year after transplantation, respectively. His S-Cr level did not significantly change from his levels 3 weeks after transplant, and was 96.3 and 93.7 μmol/L, 3 months and 1 year after transplantation, respectively. His FEUA decreased from 20.8% before the surgery, to 11.7 and 12.4%, 3 months and 1 year after transplantation, respectively. Therefore, we hypothesized the presence of donor-recipient chimerism in the tubular epithelium, played a role in the UA reabsorption process. FISH of the allograft biopsies taken 3-months and 1-year post-transplantation detected the presence of the Y chromosome in the tubular epithelium. The protocol of FISH is shown in Supplemental Material 2. Among the three biopsies, no evidence of T cell-mediated rejection, nor structural anomalies of the glomerulus, tubules, interstitium, and vessels were observed. Y chromosome-positive cells were detected mainly in the interstitium and some tubules in the graft. The interstitium typically contains white blood cells; given this, we concluded the Y chromosome-positive cells were white blood cells. Most tubular epithelial cells were of donor origin, but we also found chimeric cells (Fig. 1). After the transplantation, the donor’s S-Cr increased to approximately 88.4 μmol/L, with an FEUA of approximately 20%. She did not have any complications following the kidney donation.

## Discussion and conclusions

We reported on a kidney transplantation case in which the donated kidney had a heterozygous mutation in the *SLC22A12* (URAT1) gene, known to cause RHUC. As opposed to previously reported kidney transplants where both the donor and recipient had RHUC [7, 8], the recipient in our case did not have RHUC prior to receiving the transplanted kidney. We investigated the recipient’s physiological adaptations to receiving a kidney affected by RHUC and whether the recipient would develop RHUC.

According to a previous report, the incidence of hypouricemia, defined as an S-UA of ≤118.9 μmol/L (2.0 mg/dL), in outpatients is 0.16% in men and 0.24% in women [12]. In RHUC, urinary excretion of UA increases due to impaired membrane transport of urate in the renal proximal tubules [13]. Thus, mutations in several tubular transporters, including URAT1 and glucose transporter 9 (GLUT9), are associated with RHUC. In patients with RHUC, mutations in the *SLC22A12* gene, encoding URAT1, were the most frequent [4, 6]. The donor in our case report had a heterozygous G269A mutation in the *SLC22A12* gene. The G269A mutation is causative for RHUC and is the second most prevalent cause of RHUC [14, 15]. The allele frequency of the G269A variant is 7.3, 9.1, and 38.5% in Japanese, Koreans, and Chinese patients affected with RHUC, respectively [15].

RHUC is typically an autosomal recessive disease, caused by homozygote or compound heterozygous mutations. Moreover, single heterozygous mutations can cause hypouricemia symptoms [6, 16]. Patients with homozygous URAT1 mutations typically have an S-UA < 59.4 μmol/L (< 1.0 mg/dL), while some patients with heterozygous URAT1 mutations have an S-UA > 118.9 μmol/L (> 2.0 mg/dL) [17]. In this case, a single heterozygous mutation could cause RHUC, and both our recipient and donor had S-UA > 118.9 μmol/L. The reason why this case could be diagnosed as RHUC was owing to the donor’s status as a premenopausal woman because estrogen can accelerate UA exclusion [18].

Hypouricemia is a risk factor for urolithiasis and exercise-induced acute renal failure [2, 3]. Some patients with exercise-induced acute renal failure require temporary dialysis, and their short-term prognosis is good [2]. Only a few reports described the long-term prognosis of patients with hypouricemia, especially in patients who underwent renal transplantation [7, 8]. RHUC cases with major complications (exercise-induced acute renal failure and urolithiasis) present with UA levels < 118.9 μmol/L ([2, 17, 19, 20]. However, a few cases of exercise-induced acute renal failure have been reported with mild RHUC and with UA levels between 124.9 and 178.4 μmol/L (between 2.1 and 3.0 mg/dL) [21, 22]. Following transplantation, the recipient’s S-UA level increased (between 124.9 to 178.4 μmol/L), the FEUA level decreased, and the S-Cr level did not change. The stable S-Cr level, in particular, is a sign of a good outcome. It was important to follow-up with this patient to monitor for potential hypouricemia complications. Moreover, the patient was directed to avoid strenuous exercise, which may lead to exercise-induced acute renal failure, and to drink plenty of water, which will alkalize his urine and help prevent urolithiasis [23, 24].

We speculated that the increase in S-UA and decrease in FEUA levels were owing to an adaptive physiological change after transplantation. In contrast with previous reports in which both the recipient and the donor were affected by RHUC, in our case report, only the donor had the disease [7, 8]. Therefore, we suspected the presence of chimerism in the tubular epithelium. As expected, the allograft biopsies taken 3-months and 1-year post-transplantation and evaluated by FISH showed the presence of cells carrying the Y chromosome in the tubular epithelium. Sex chromosome chimerism, particularly Y chromosome chimerism, is common in kidney allograft biopsies from sex-mismatched pairs and is detected in approximately 40–70% of kidney biopsies in both genders performed more than 1-year post-transplantation [9]. To the best of our knowledge, this is the first report identifying chimerism in tubular epithelial cells after kidney transplantation from a donor affected by RHUC to a recipient without RHUC. We hypothesize that the serum-UA level in the recipient increased owing to the chimerism of the tubular epithelium.

This case report had an important limitation. Although we speculated that the decrease in FEUA level observed in the recipient was related to tubular chimerism, other factors may have been responsible for this decrease, especially since the ratio of tubular chimerism was very small. We estimated that the ratio was around 10%, but we did not have enough images to perform a semi-quantitative analysis. Moreover, although the clinical course of the recipient was good in the short-term, the recipient and donor have to be monitored very closely, especially for signs of renal failure.

In conclusion, we reported on a kidney transplantation case in which the donor was affected by RHUC due to a heterozygous mutation in the *SLC22A12* (URAT1) gene, and the recipient did not develop RHUC owing to the presence of chimerism in the tubular epithelium. Further studies are necessary to predict the clinical outcomes following kidney transplantation for donors and recipients when one or both are affected by RHUC.

## Supplementary information

**Additional file 1: Supplemental Material 1.** The American College of Medical Genetics and Genomics variant classifications variant classifications to (NM_144585.4:c.269G > A:p.(Arg90His).

**Additional file 2: Supplemental Material 2.** FISH protocol.

## Data Availability

Anonymized data can be provided by the corresponding author for a reasonable request.

## Abbreviations

RHUC: Renal hypouricemia
FISH: Fluorescence in situ hybridization
FEUA: Fractional excretion of uric acid
URAT1: The urate transporter 1
