# Supplementary material for: Transplantation of a kidney with a heterozygous mutation in the SLC22A12 (URAT1) gene causing renal hypouricemia: a case report
Source: BMC Nephrol. 2020 Jul 16;21:282. doi: 10.1186/s12882-020-01940-4 (PMC7364597; doi:10.1186/s12882-020-01940-4)
Supplement: Supplementary file 1 — Additional file 1: Supplemental Material 1. The American College of Medical Genetics and Genomics variant classifications variant classifications to (NM_144585.4:c.269G > A:p.(Arg90His). [file 12882_2020_1940_MOESM1_ESM.docx]

**Supplemental Material 1. The American College of Medical Genetics and Genomics variant classifications variant classifications to (NM_144585.4 :c.269G>A :p.(Arg90His)**

The variant classification is “Uncertain significance”.

PM2 (moderate)

PP2 (supporting)

PP5 (supporting)

BP1 (supporting)
