# Supplementary material for: Transplantation of a kidney with a heterozygous mutation in the SLC22A12 (URAT1) gene causing renal hypouricemia: a case report
Source: BMC Nephrol. 2020 Jul 16;21:282. doi: 10.1186/s12882-020-01940-4 (PMC7364597; doi:10.1186/s12882-020-01940-4)
Supplement: Supplementary file 2 — Additional file 2: Supplemental Material 2. FISH protocol. [file 12882_2020_1940_MOESM2_ESM.docx]

**Supplemental Material 2. FISH protocol**

***Probe***

Human XY chromosome FISH probes (Y: FITC, X: Cy3) were acquired from Chromosome Science Labo Inc.

***Pretreatment***

Wash slides in PBS at RT (approximately 25 ºC).

Dehydrate using alcohol series (70% and 100%) and dry.

***Hybridization***

Apply 10 μL of probe and cover by coverslips.

Denature at 90 ºC for 10 min using a hotplate.

Hybridize overnight at 37 ºC in a wet chamber.

***Washing***

Keep hybridized slides in 2x SSC solution (0.03M Sodium citrate, 0.3M NaCl, pH: 7.0) for 5 min and remove coverslips gently.

Keep slides in 50% formamide/2xSSC for 20 min at 37 ºC. SCC, saline sodium citrate

Keep slides in 1x SSC solution (0.015M Sodium citrate, 0.15M NaCl, pH: 7.0) for 15 min at RT (approximately 25 ºC) (hapten-labeled probes only).

***Counterstain***

Stain with DAPI and mount with an antifade mounting medium.
